# Treatment of Intracranial Pseudoaneurysms With a Novel Covered Stent: A Series of 19 Patients With Midterm Follow-Up

**DOI:** 10.3389/fneur.2020.580877

**Published:** 2020-11-25

**Authors:** Wei Wang, Xihong Liang, Guangli Chen, Peng Yang, Jialiang Zhang, Haocheng Liu, Shangfeng Zhao, Yong Li, Bowen Sun, Jun Kang

**Affiliations:** Neurosurgery Department, Tongren Hospital of Capital Medical University, Beijing, China

**Keywords:** pseudoaneurysms, covered stent, endoleak, feasibility, outcome

## Abstract

**Background:** The optimal treatment for intracranial pseudoaneurysm is unclear. This study aims to analyze the outcome of treating intracranial pseudoaneurysm with a novel covered stent.

**Materials and Methods:** The institutional imaging and clinical databases were retrospectively reviewed for patients with intracranial pseudoaneurysms treated with Willis covered stent from January 2017 to December 2019. The clinical presentations, etiology, intraoperative complications, and immediate and follow-up outcomes were analyzed.

**Results:** A total of 19 patients with 20 pseudoaneurysms were enrolled for analysis. Seventeen patients presented with vision loss and two with epistaxis. Nineteen Willis covered stents were used with one for each patient without technical failure. Intraoperative thrombosis was encountered in one patient (5.3%), which was recanalized by tirofiban. During clinical follow-up, no further epistaxis occurred, and visual acuity improved in three (17.6%) patients. Endoleak occurred in seven (36.8%) patients after the initial balloon inflation and persisted in one (5.3%) patient after balloon re-inflation. This endoleak disappeared at 8 month follow-up. Finally, during angiographic follow-up (median 13 months), parent artery occlusion and in-stent stenosis occurred in one (5.3%) patient. No stent-related ischemic event was encountered.

**Conclusions:** The Willis covered stent is feasible, safe, and efficient in treating intracranial pseudoaneurysms.

## Introduction

Pseudoaneurysms are characterized by complete disruption of an arterial wall, resulting in an extravascular hematoma contained by a thin layer of connective tissue (1, 2). The possible etiology of intracranial pseudoaneurysms includes traumatic and iatrogenic injury, radiation therapy, vasculitis, infection, and rupture of a true saccular aneurysm or arteriovenous malformations (2, 3). Traumatic intracranial pseudoaneurysms account for <1% of patients with cerebral aneurysms (1). Most pseudoaneurysms will not regress spontaneously but will rupture within 3 weeks, causing a mortality rate ranging from 31 to 54% (1, 4). A previous report has demonstrated three times less of death for patients diagnosed before aneurysm rupture, compared with those diagnosed after rupture (5). Delayed diagnosis can contribute to the high morbidity and mortality of patients with these lesions (6). The prompt diagnosis and proper treatment are necessary for this disease.

A triad of symptoms has been proposed as indications for intracranial traumatic pseudoaneurysms, which include massive epistaxis, unilateral blindness, and skull base fracture (7). Delayed repeated epistaxis after head and facial trauma is a distinctive manifestation of intracranial traumatic pseudoaneurysms (6). Other clinical manifestations include unilateral vision loss, cerebral ischemia, or compressive effect. Although computed tomography (CT) and magnetic resonance imaging can provide valuable indications (8), digital subtraction angiography (DSA) remains the gold standard for diagnosis (6).

The primary objective in the treatment of pseudoaneurysm is the complete obliteration of the lesion from the parent artery. Recent decades have witnessed a transmission from microsurgical to endovascular treatment for traumatic intracranial pseudoaneurysms, with increased parent artery preservation and decreased morbidity and mortality (9). Destructive strategies include aneurysm trapping by parent artery ligation or endovascular occlusion with coils or balloons, with or without bypass surgery (10–13). The relatively high mortality and morbidity have rendered destructive treatment from the first-line choice (10–13). Constructive strategies, including aneurysm coiling with coils, balloons, or liquid embolization agents with or without stent or balloon assistance, have been widely reported (3, 9, 14–16). The disadvantages of these approaches include technical sophistication, high risk of aneurysm rupture for the manipulation of the coils and micro-catheter within the friable aneurysmal lumen, and long-term durability inherent in coiling approaches (2). Some cases have been treated with flow diverters. However, the antiplatelet therapy and delayed occlusion of the aneurysm after flow diverter implantation may leave the patient at risk for aneurysm rupture before complete occlusion (17, 18).

Theoretically, covered stents are ideal for intracranial aneurysms as the immediate exclusion of aneurysm from the circulation. Many authors have reported their initial experience with covered stents for intracranial pseudoaneurysms (3, 6, 19–21). A total of five types of covered stents have been reported in the literature, of which only the Willis (MicroPort Medical Co. Shanghai, China) covered stent is exclusively designed for intracranial vasculatures (20, 21). The stiffness and risk of perforator coverage are the primary disadvantages for covered stents in treating intracranial pseudoaneurysms (2, 20, 21). As a novel device exclusive for intracranial vasculatures, the Willis covered stent is designed to be compatible with the size of the internal carotid artery, and the abundance in stent length provides the feasibility of selecting a stent with minimal length to cover the vascular defect, thereby decreasing the difficulties in delivery and risks of perforator coverage (21). Although the Willis covered stent shows potential application in treating intracranial pseudoaneurysms, the reported cases are limited, and the long-term outcome remains unclear (20, 21).

In this study, we reported a series of 19 patients with 20 intracranial pseudoaneurysms treated with the Willis covered stent. The demographic information, etiology, clinical presentations of the patients, intraoperative complications, and immediate and follow-up clinical and angiographic outcomes were summarized and discussed.

## Materials and Methods

### Patient Cohort

This study was approved by the Institutional Ethic Committee of Tongren Hospital affiliated to Capital Medical University, and written informed consents were obtained from all patients or their legal guardians for those younger than 18 before operation. All patients enrolled in this study were treated with the Willis covered stents in our institution from January 2017 to December 2019, with a definite diagnosis of intracranial pseudoaneurysms. Pseudoaneurysms of the extracranial internal carotid artery were excluded for analysis.

### Intervention Procedure

All patients received Willis covered stent implantation under general anesthesia. In brief, a 6F Envoy guiding catheter (Cordis, Miami Lakes, FL) was positioned in the targeted internal carotid artery through a right femoral approach. Control angiograms were performed to illustrate the details of the aneurysm and select the appropriate stent version. A balloon occlusion test (BOT) was conducted to evaluate the collateral circulations as previously reported (6). A 0.014 inch Transcend microwire (300 cm, Transcend Floppy; Boston Scientific, Natick, Mass) was delivered to the distal branches of the ipsilateral middle cerebral artery using a microcatheter (Echelon-10, eV3 Neurovascular, Inc. USA). Then, the microcatheter was withdrawn, and the Willis covered stent was delivered along the microwire to cover the orifice of the aneurysm on the basis of the roadmap. After a control angiography, which confirms the position of the stent, the balloon was inflated at 6 atmospheric pressure to deploy the stent. An immediate angiogram was performed to evaluate the imaging results. If an endoleak was detected, then the balloon was inflated two times. If the endoleak disappeared or diminished to a slight flow volume, then the procedure would be ceased. Otherwise, another stent would be considered.

Before the procedure, aspirin (75 mg/day, Bayer AG, Germany) and clopidogrel (100 mg/day, Plavix, Sanofi-Synthelabo, France) were prescribed for each patient for at least 3 days. For emergent operation, 300 mg of aspirin and clopidogrel each were taken orally 2 h before the procedure. A bolus of 5,000 IU heparin was injected transvenously after the introduction of the femoral sheath, followed by 2,000 IU in the next hour and 1,000 IU for each hour thereafter, to maintain an activated clotting time of 250 to 300 s. Heparin was given for 48 h after the procedure, and aspirin and clopidogrel were taken together for 3 months, followed by aspirin alone for another 3 months.

### Postoperative Evaluation

CT scanning was routinely arranged the next day after the operation to evaluate hemorrhagic or ischemic complications. The clinical conditions were evaluated every day after the operation until discharge. The first clinical interview was arranged 1 month after the operation, and angiographic follow-up was arranged 6 to 12 months after the procedure.

The clinical and angiographic assessment was in accordance with the previous report (21). The resistance force in delivering the stent to the target vessel was ranked into (1) no resistance, (2) no apparent resistance, (3) evident resistance but could be overcome, and (4) extreme resistance for the failure of delivery. The apposition of the Willis covered stent to the vascular wall after deployment was categorized as (1) no endoleak, (2) minor endoleak with the delayed evacuation of the aneurysm sac, and (3) apparent endoleak that caused synchronized visualization of the aneurysm sac with the parent artery. Clinical evaluation of the symptoms was categorized into (1) complete recovery, (2) improvement, (3) no change, and (4) deterioration.

## Results

### Patient Information and Aneurysm Characteristics

A total of 20 intracranial pseudoaneurysms in 19 patients were enrolled in this study. Eighteen patients had a single aneurysm, and one patient had two aneurysms (Case 8). The demographic information of the patients and the characteristics of the aneurysms were summarized in Table 1. Of the 19 patients, five (26.3%, 5/19) were females, and the rest (73.7%, 14/19) were males. The mean age of the patients was 30.9 ± 12.6 years, ranging from 11 to 63 years. With regard to the etiology of the aneurysms, the majority (63.2%, 12/19) of the aneurysms resulted from a traffic accident, followed by fall-down injury (26.3%, 5/19). Iatrogenic and stabbing injury resulted in one (5.3%, 1/19) pseudoaneurysm in our series. The majority of the patients referred to the institution with the chief complaint of visual loss (89.5%, 17/19), followed by epistaxis (10.5%, 2/19). Sixteen (84.2%, 16/19) patients were initially diagnosed by CT angiography, and three (15.8%, 3/19) patients encountered massive bleeding during endoscopic optic canal decompression. Carotid-cavernous fistula (CCF) accompanied by pseudoaneurysm was observed in four patients (21.1%, 4/19). All CCFs had low volume, and their presentation was occult, with slight chemosis in all four patients. No typical signs of CCF such as exophthalmos and intracranial bruit were detected. Skull base bone fracture was found in 94.7% (18/19) of all the patients.

**Table 1:** Demographic, clinical presentation, endovascular treatment, and follow-up outcomes of the patients.

**Patient No./Age/Sex**	**Clinical presentations**	**Etiology**	**Accompanied with CCF/optic nerve injury/skull base fracture**	**Diagnosis delay**	**Aneurysm location**	**Aneurysm size/neck (mm)**	**Stent version (mm X mm)**	**Immediate angiographic result**	**Final clinical follow-up time (m)/result**	**Final angiographic follow-up/regime** **/time (m)/result**
1/22/M	Vision loss	Accident	No/Yes/Yes	14 days	L/C5	3*5/3	4.0 X 16	Minor endoleak, radiation, disappear	48/No change	CTA/12/Patent
2/44/M	Vision loss	Fall	No/Yes/Yes	3 days	L/C4	2*3/2	4.5 X 13	Apparent endoleak, redilation, disappear	13/No change	CTA/13/Patent
3/23/M	Vision loss	Accident	No/Yes/Yes	30 days	R/C5	1*2/2	4.0 X 16	Good	12/No change	CTA/10/Patent
4/44/M	Vision loss	Fall	Yes/No/Yes	2 days	L/C4	10*10/7	4.0 X 16	Good	6/No change	DSA/2/Occlusion
5/37/M	Epistaxis	Accident	No/No/No	16 days	R/C4	2*5/3	4.5 X 7	Good	6/No further epistaxis	CTA/6/Good
6/35/F	Vision loss	Iatrogenic	No/Yes/Yes	3 days	L/C6	4*5/4	3.5 X 10	Minor endoleak, redilation, disappear	12/No change	CTA/8/Good
7/11/F	Vision loss	Fall	Yes/Yes/Yes	5 days	R/C4	4*5/3	3.5 X 13	Good	12/Improvement	CTA/10/Good
8/28/F	Vision loss	Accident	No/Yes/Yes	29 days	R/C4	2*2/2; 4*5/4	3.5 X 16	Good	13/No change	CTA/13/Slight in-stent stenosis
9/18/F	Vision loss	Accident	No/Yes/Yes	10 days	L/C4	5*7/5	3.5 X 13	Good	14/ No change	CTA/14/Good
10/22/F	Vision loss	Accident	No/Yes/Yes	2 days	L/C4	4*5/3	4.5 X 13	Endoleak	8/Improvement	CTA/6/Patent
11/50/M	Vision loss	Accident	No/Yes/Yes	31 days	L/C4	3*5/4	3.5 X 13	Minor endoleak, redilation, disappear	8/No change	CTA/8/Good
12/37/M	Vision loss	Accident	No/Yes/Yes	7 days	R/C6	3*5/3	3.5 X 10	Good	12/No change	DSA/12/Good
13/63/M	Vision loss	Fall	No/Yes/Yes	8 days	R/C4	2*4/2	4.0 X 10	No endoleak, A1 embolism	12/No change	CTA/12/Good
14/26/M	Vision loss	Accident	Yes/Yes/Yes	5 days	R/C4	2*3/2	4.0 X 16	Good	11/No change	CTA/11/Good
15/38/M	Vision loss	Sharp instrument Injury	Yes/Yes/Yes	6 days	L/C4	5*7/4	4.0 X 13	Minor endoleak, redilation, disappear	10/No change	CTA/10/Good
16/18/M	Vision loss	Fall	No/Yes/Yes	13 days	L/C4	20*40/9	4.0 X 13	Good	9/No change	CTA/6/Good
17/36/M	Vision loss	Accident	No/Yes/Yes	1 days	R/C4	4*5/4	4.0 X 10	Good	7/No change	DSA/7/Good
18/21/M	Vision loss	Accident	No/Yes/Yes	20 days	R/C4	2*5/3	4.0 X 10	Minor endoleak, redilation, disappear	9/Improvement	DSA/6/Good
19/25/M	Epistaxis	Accident	No/Yes/Yes	60 days	R/C4	4*6/5	4.5 X 13	Good	6/No further epistaxis	DSA/5/Good

*L, left; R, right; CCF, carotid-cavernous fistula; A, anterior cerebral artery; CTA, computed tomography angiography; DSA, digital subtraction angiography*.

With regard to aneurysm characteristics, nine (45.0%, 9/20) were located in the left internal carotid artery, and 11 (55.0%, 10/19) were located in the right internal carotid artery. Sixteen (80.0%, 16/20) aneurysms were located at the cavernous segment (C4) of the internal carotid artery, and two (10.0%, 2/20) aneurysms were located at the clinic (C5) and ophthalmic (C6) segment. The median maximum diameter of the aneurysms was 5 mm (ranging from 2 to 40 mm), and the median neck was 3 mm (ranging from 2 to 9 mm).

### Immediate Results

All procedures were technically successful without aneurysm rupture and artery perforation. Intraoperative embolic complication was encountered in one (5.3%, 1/19) patient (Patient No. 13), in whom the ipsilateral A1 was occluded by thrombosis. Tirofiban hydrochloride (Lunan Pharmaceutical Group, Linyi, China) was injected trans-arterially as a bolus (0.25 mg), and control angiography for 15 min showed recanalization of the affected artery. Tirofiban hydrochloride was maintained (0.2 mg/h) for the following 48 h.

A total of 19 stents were used with one stent for each patient, and no coils were packed for occluding the aneurysms. The versions of the stents were demonstrated in Table 1. Nine stents had a nominal diameter of 4 mm; six were 3.5 mm, and four were 4.5 mm. With regard to the length of these stents, eight were 13 mm; five were 16 mm; five were 10 mm, and one was 7 mm. During delivery, no resistance was encountered in nine procedures. However, minor and apparent resistance was observed in eight and two procedures, respectively. Of the four patients with CCFs, three were covered by the Willis stent simultaneously with the pseudoaneurysm. In Patient No. 7 (illustrative case 2, **Figure 2**), CCF was detected in the first angiography, but the drainage vein occluded after ipsilateral carotid compression, resulting in pseudoaneurysm. This pseudoaneurysm was treated with a Willis stent.

After the initial stent deployment, 13 aneurysms in 12 patients (63.2%, 12/19) disappeared immediately. Endoleak occurred in seven (36.8%, 7/19) patients, of which six (31.8%, 6/19) were minor and one (5.3%, 1/19) was apparent (Patient No. 2). Second balloon inflation sealed six (85.7%, 6/7) endoleaks, and endoleak persisted in one patient (Patient No. 10, illustrative case 4, **Figure 4**).

### Angiographic Follow-Up

Plain CT scanning was routinely arranged for each patient the next morning after the operation. No evident hemorrhage or infarction was detected in all patients. All patients received angiographic follow-up, of which 14 received at least one CTA follow-up, and five patients received DSA. The mean final angiographic follow-up time was 9 ± 3.32 months, ranging from 2 to 14 months. The angiographic results revealed that all aneurysms were completely treated with slight in-stent stenosis in one patient (Patient No. 8), and parent artery occlusion was encountered in one patient (Patient No. 4), in whom the dual antiplatelet therapy was discontinued for about 30 days after the procedure. This patient tolerated this occlusion well without any ischemic attack.

### Clinical Follow-Up

Clinical follow-up was conducted every day after the operation and before discharge. Outpatient follow-up was routinely arranged for all patients at 1 and 6 months after the operation. The median last clinical follow-up time was 11 months, ranging from 6 to 48 months (Table 1). Epistaxis was never encountered in the two patients initially presented with epistaxis. Visual acuity gradually improved in three (17.6%, 3/17) patients and was stable in the 14 (82.4%, 14/17) patients. In the four patients accompanied by CCF, chemosis gradually diminished after the operation and was completely resolved at 1 month outpatient follow-up.

### Illustrative Cases

#### Case 1 (Patient No. 2)

**Figure 1:**
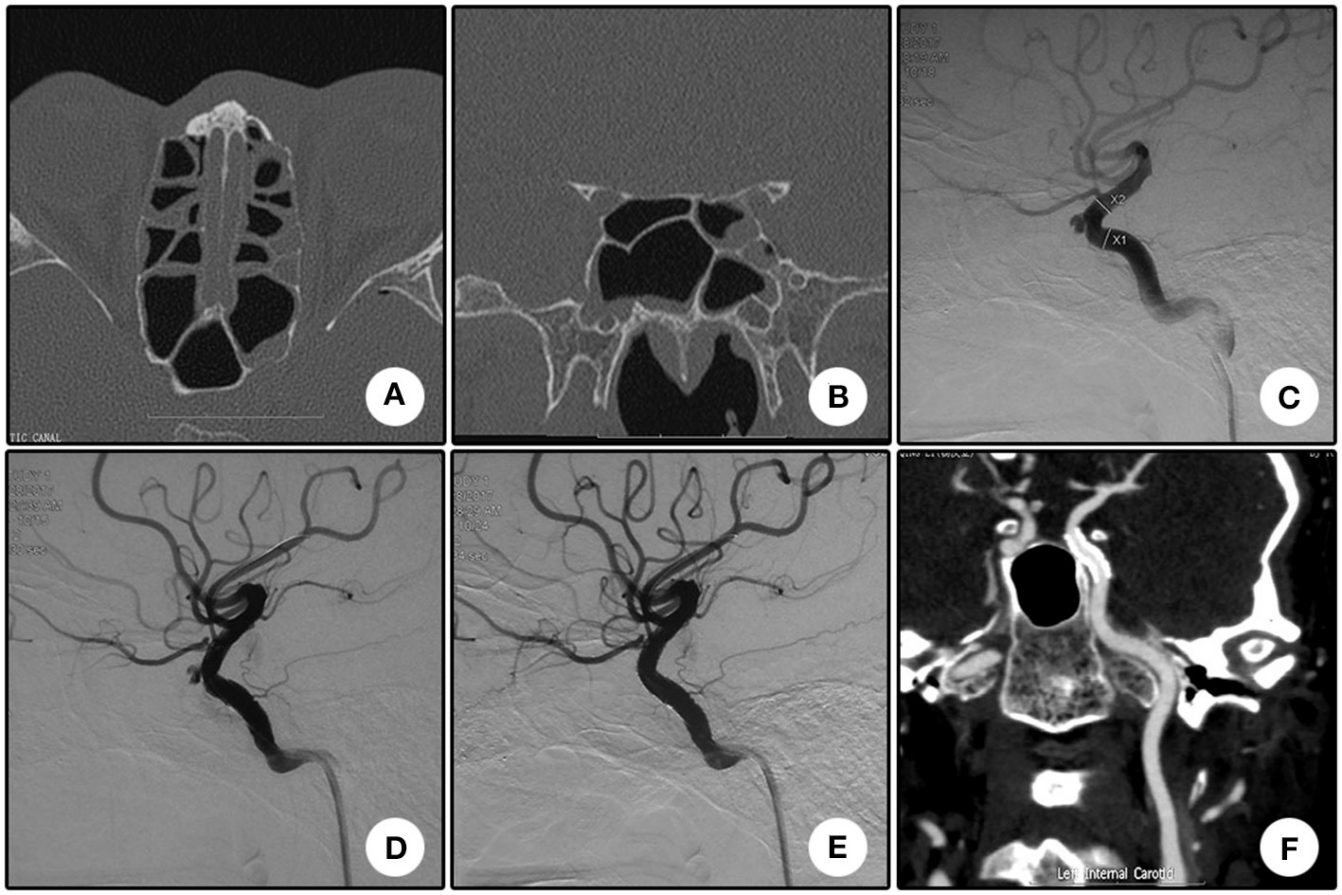
Illustrative case 1. **(A)** Sectional CT of the bone window revealed the bone fracture of the posterior portion of the left lateral wall of the sphenoidal sinus and the submucosal hemorrhage. **(B)** Coronary CT revealed the bone fracture of the left lateral wall of the sphenoidal sinus and the submucosal hemorrhage. **(C)** DSA showed a pseudoaneurysm of the left cavernous internal carotid artery. **(D)** Apparent endoleak after the initial stent deployment. Note the change of the siphon curve. **(E)** The endoleak disappeared after the second balloon inflation. The siphon curve became straighter. **(F)** CT angiography 13 months after the operation showed patency of the parent artery without stenosis.

This patient was a 44 year-old man. He was transferred to our department for the chief complaint of left vision loss after a fall-down injury for 2 days. CT scanning showed bone fractures of the left optic canal at the inferior medial wall with local submucosal hemorrhage (Figures 1A,B). The emergent trans-endoscopic optic nerve canal decompression was performed to save his left vision. During the operation, after removing the mucosa adjacent to the internal carotid recess, arterial reddish blood ejected. A pseudoaneurysm of the cavernous carotid artery was suspected, and the bleeding was ceased with nasal packing without further optic nerve canal decompression. The next morning, a DSA examination confirmed the diagnosis of a pseudoaneurysm (Figure 1C), and a Willis stent was planned. After the initial balloon inflation to deploy the stent, an apparent endoleak was encountered (Figure 1D). A second balloon dilation completely sealed the endoleak and reshaped the curve of the carotid siphon (Figure 1E). The nasal packing was withdrawn the next morning after the procedure with no bleeding. No bleeding was encountered during a 13 month clinical follow-up. During CTA follow-up 13 months after the procedure, patency of the parent artery was observed (Figure 1F), but his left vision had no improvement.

#### Case 2 (Patient No. 7)

**Figure 2:**
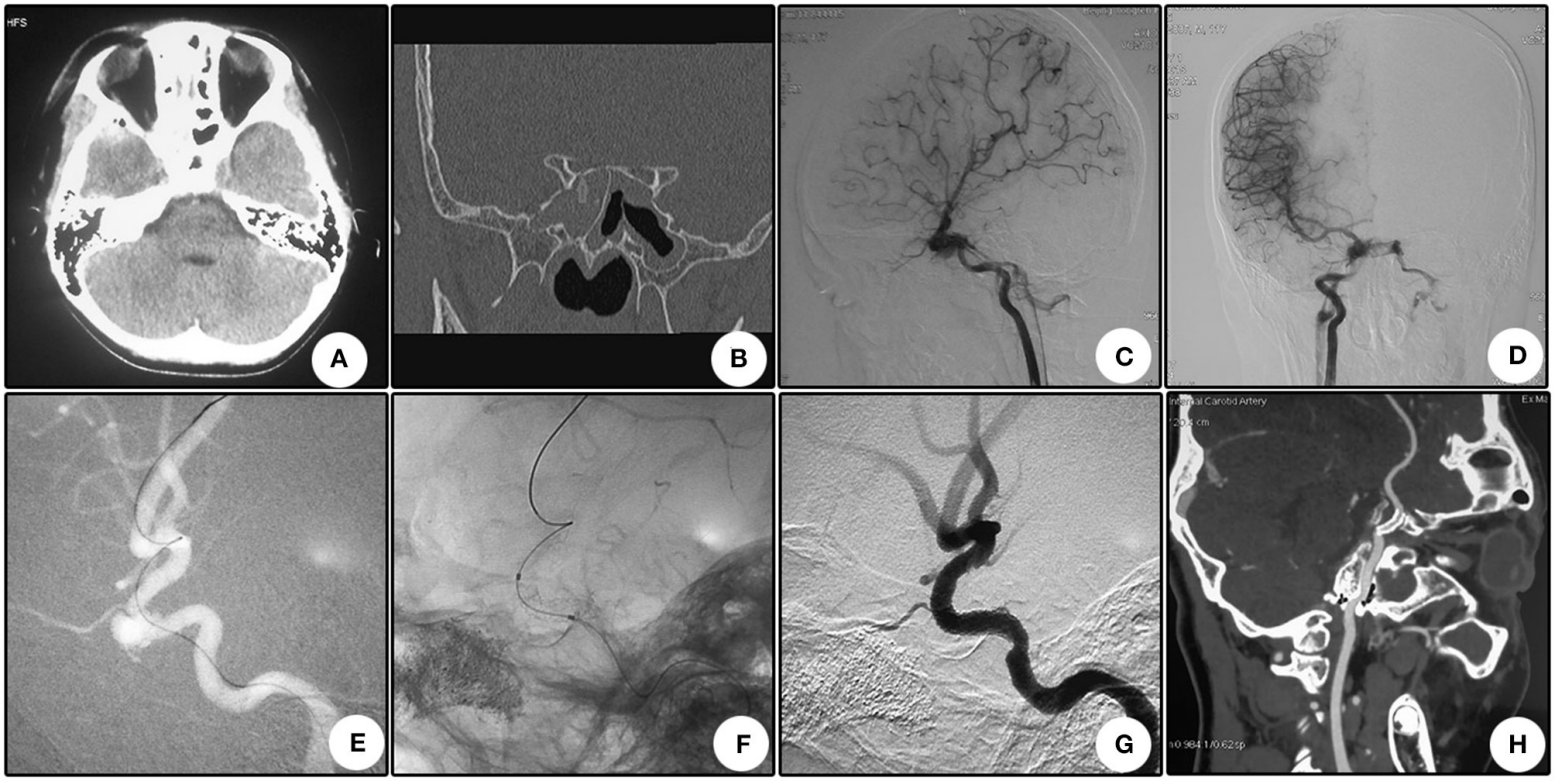
Illustrative case 2. **(A)** CT scanning immediately after the injury showed right temporal contusion and intracranial pneumatocele. **(B)** Coronary CT showed the bone fracture of the optic nerve canal. **(C,D)** Lateral **(C)** and anterior **(D)** view of the right internal carotid artery angiography. The right internal carotid-cavernous fistula was confirmed and drained via bilateral inferior petrosal sinuses. **(E)** After common carotid artery compression for 12 days, angiography revealed closure of the drainage and formation of a pseudoaneurysm. **(F)** Apposition of the stent to the artery lumen. **(G)** The pseudoaneurysm disappeared after stent deployment, and the curve of the siphon became straighter. **(H)** CTA follow-up 10 months after the operation revealed the patency of the parent artery with no evident stenosis.

An 11 year-old girl who suffered from right vision loss after a fall-down injury for 4 days was transferred to our department. CT scanning showed right temporal contusion, intracranial pneumatocele, and bone fractures of the right optic nerve canal (Figures 2A,B). Emergent trans-endoscopic right optic nerve canal decompression was conducted, and the intraoperative hemostasis for the cavernous sinus was more difficult than previous procedures. Combined with the sign of right chemosis, a CCF was highly suspected. DSA, which was conducted the next day after the decompression operation, showed a low-volume CCF drained by the bilateral inferior petrosal sinus (Figures 2C,D). Ipsilateral common carotid artery compression was conducted two times a day for 12 days. The right chemosis gradually vanished, and during DSA follow-up, the disappearance of the drainage and formation of a cavernous pseudoaneurysm were observed (Figure 2E). After the deployment of a Willis covered stent, the pseudoaneurysm disappeared (Figures 2F,G). During CTA follow-up, the patency of the parent artery showed no evident stenosis (Figure 2H). Furthermore, during clinical follow-up, a dramatic improvement of her right vision was confirmed.

#### Case 3 (Patient No. 8)

**Figure 3:**
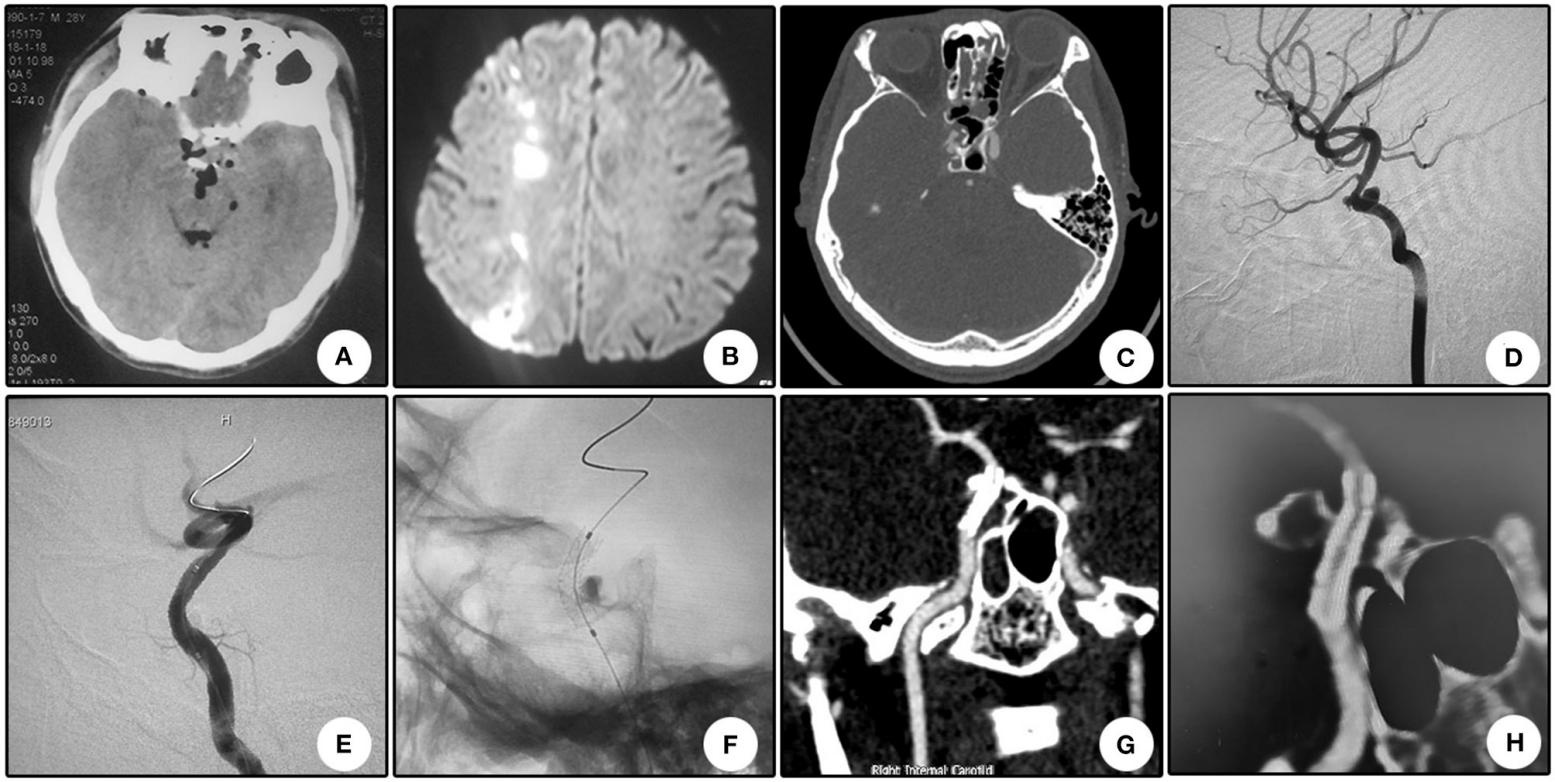
Illustrative case 3. **(A)** CT scanning immediately after the injury showed multiple skull bone fracture and intracranial pneumatocele. **(B)** Magnetic resonance imaging 7 days after trauma showed hypoperfusion infarction of the right hemisphere. **(C)** Sectional CT with arterial contrast revealed irregularity of the right internal carotid artery and adjacent bone fractures of the lateral wall of the sphenoidal sinus. **(D)** DSA revealed two pseudoaneurysms located oppositely at the cavernous internal carotid artery. **(E)** After stent deployment, these two pseudoaneurysms disappeared. **(F)** Apposition of the stent to the artery wall. **(G)** CTA 8 days after operation showed patency of the parent artery. **(H)** CTA 13 months after operation showed patency of the parent artery and slight in-stent stenosis as indicated by the relative minimal flow distal to the stent.

This patient suffered from bilateral vision loss for 28 days after a severe traffic accident. Immediate CT scanning showed multiple skull bone fractures and intracranial pneumatocele (Figure 3A). Seven days after the head trauma, he encountered right cerebral hypoperfusion infarction (Figure 3B). Sectional CTA imaging 28 days after the trauma demonstrated bilateral wall bone fractures of the sphenoidal sinus and irregularity of the right cavernous carotid artery (Figure 3C). DSA revealed two pseudoaneurysms located oppositely at the right cavernous carotid artery (Figure 3D). After a Willis stent implantation, these two aneurysms disappeared simultaneously (Figures 3E,F). CTA 8 days after the operation showed patency of the parent artery (Figure 3G). CTA 13 months after the operation showed patency of the parent artery and slight in-stent stenosis as indicated by the relative minimal flow distal to the stent (Figure 3H). During a 13 month clinical follow-up, no further ischemic attack was encountered, but no improvement of bilateral vision was observed.

#### Case 4 (Patient No. 10)

**Figure 4:**
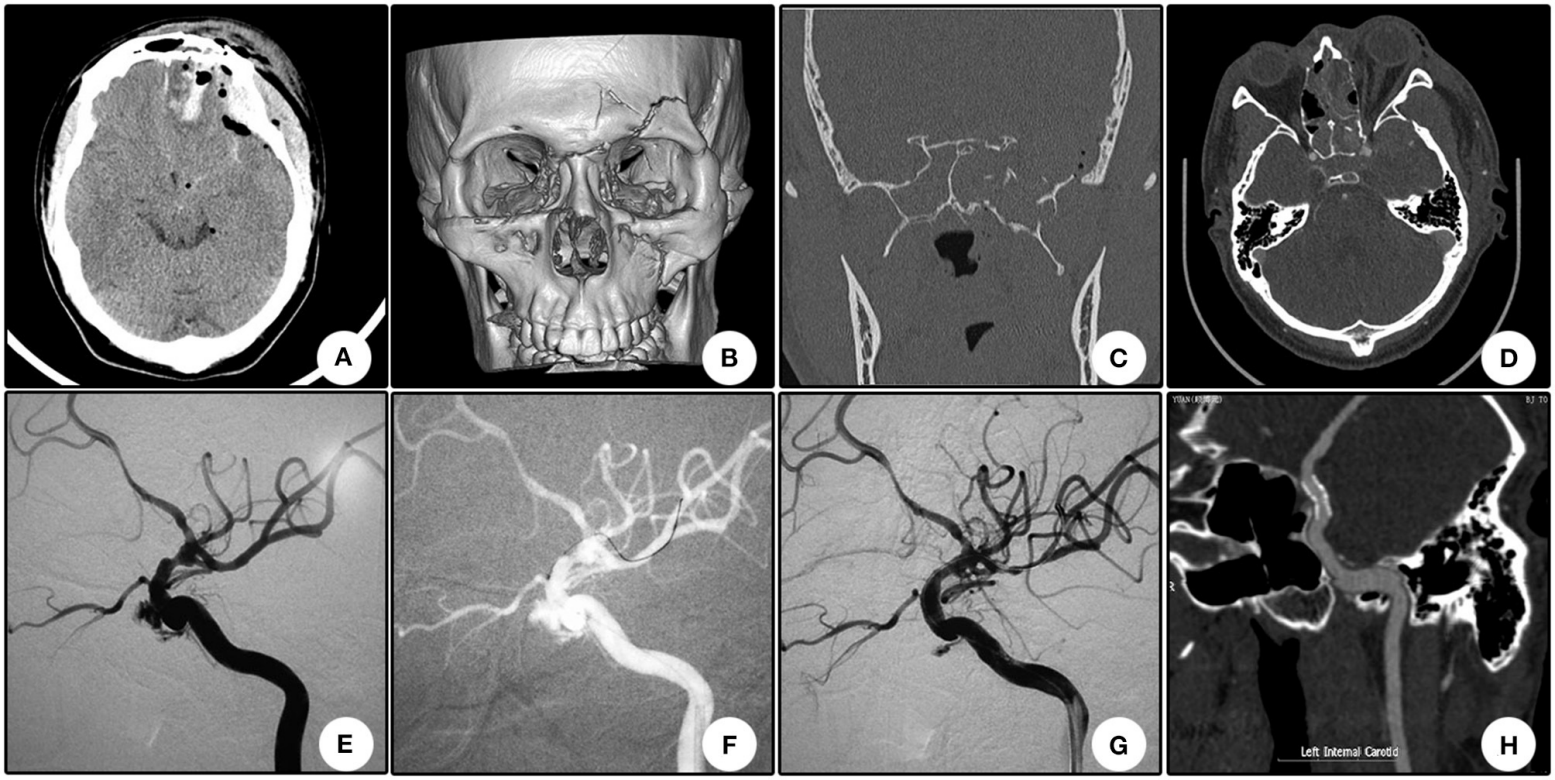
Illustrative case 4. **(A)** CT scanning immediately after trauma showed multiple skull bone fracture, right frontal contusion, and pneumatocele. **(B)** Sectional CT with arterial contrast revealed irregularity of the left internal carotid artery and adjacent bone fractures of the lateral wall of the sphenoidal sinus. **(C)** Coronary CT showed the massive bone fracture of the left lateral wall of the sphenoidal sinus. **(D)** Three-dimensional reconstruction of the skull showed multiple bone fractures of the lateral wall of the left orbit. **(E)** DSA showed the pseudoaneurysm of the left cavernous internal carotid artery. **(F)** Position confirmation of the stent. Note the two markers indicating the ends of the stent. **(G)** After the initial deployment and second balloon dilation, a minor endoleak remained. **(H)** CTA 6 months after the operation showed patency of the parent artery and no signs of the endoleak.

A 22 year-old boy who suffered from left vision loss and rhinorrhea for 11 h after a vehicular accident was referred to our department on September 5, 2018. Initial CT showed multiple skull bone fractures and pneumatocele (Figure 4A). He obtained nasal packing for rhinorrhea in a local hospital. After admission, CT reconstruction revealed multiple bone fractures of the lateral wall of the left orbit (Figure 4B) and left lateral wall of the sphenoidal sinus (Figure 4C). The nasal packing was removed the next day after admission, and conservative treatment was conducted for the cerebral spinal fluid leakage. Sectional CTA imaging was performed on September 7, which revealed lumen irregularity of the initial part of the cavernous internal carotid artery (Figure 4D). On September 13, DSA confirmed the diagnosis of left internal carotid artery pseudoaneurysm of the cavernous segment (Figure 4E). A Willis stent was delivered to cover the orifice of the aneurysm, but minor endoleak was encountered even after a second balloon dilation (Figure 4F). During CTA follow-up 6 months after the operation, complete healing of the aneurysm and patency of the parent artery without in-stent stenosis were observed (Figure 4H). During clinical follow-up, the cerebral spinal fluid leakage was completely healed, but no improvement of his left eye vision was observed.

## Discussion

The Willis covered stent system is the only covered stent system designed exclusively for intracranial vasculatures. In this study, a consecutive series of 20 intracranial pseudoaneurysms in 19 patients was treated with this novel device. Based on previous reports, this study is the largest case series of using the covered stent for intracranial pseudoaneurysms. Our results indicated the safety and efficacy of this device for intracranial pseudoaneurysms.

Recent decays have encountered a trend from deconstructive treatment to reconstructive treatment for intracranial pseudoaneurysms. Deconstructive strategies referred to parent artery sacrifice by either microsurgical or endovascular trapping of the aneurysm, with or without extracranial and intracranial bypass (17). Several studies have proven the efficacy and durability of this technique (6, 10). Ischemic events are the main concerns for occluding the parent internal carotid artery; thus, a BOT is mandatory to evaluate the contralateral circulation (6, 22). However, even in patients that tolerated a BOT, 5 to 22% would develop ischemic complications or cerebral infarcts (11–13). Another concern is aneurysm formation because of the hemodynamic effects after internal carotid artery occlusion (23, 24). For patients who failed to tolerate the BOT, bypass surgery could be considered. However, these procedures are technically demanding and associated with a substantial morbidity rate (25).

With the development of endovascular devices, reconstructive strategies with patent artery conservation have been widely reported, including coil packing with or without stent/balloon assistance (3, 14, 26), liquid agent embolization (9, 16), bare stent implantation (22), and covered stent/flow diverter implantation (3, 17, 18, 20, 21, 27, 28). Direct aneurysm sac packing with coils, balloons, or liquid agents carries a high risk of aneurysm rupture because of the fragile aneurysm wall (15). Furthermore, recurrence is relatively common in pseudoaneurysms with packing, either with coils or liquid agents (3, 9, 21, 26, 29). Dual antiplatelet therapy is mandatory after stent implantation, increasing the risk of aneurysm rupture before complete intra-aneurysmal thrombosis (15, 22). Recently, flow diverter has been applied for intracranial pseudoaneurysm in case reports (17, 18, 27). The main limitation of a flow diverter is also the delayed thrombosis of the aneurysm sac, which leaves a potential risk of rebleeding during this interim (17, 18).

Covered stent seems ideal for immediate repair of the vascular defect without direct intra-aneurysmal intervention. Many authors have reported their successful cases with covered stents (3, 6, 19–21, 28). Before the Willis stent, four types of covered stents had been reported: autologous vein-covered stents, the Symbiot covered stent (Boston Scientific), the Jostent covered stent (Abbott Vascular, Redwood City, CA), and the carotid Wall stent (Boston Scientific) (20). The application of these four types of stents is off label and carries the common disadvantages, such as size mismatch, delivery difficulty, perforator coverage, and the potential risk of vessel damage from the high-pressure release (6, 20, 22). The Willis covered stent is designed exclusively for intracranial vasculature, aiming to address the inherent disadvantages from the abovementioned covered stents (20). First, the abundance in versions with various diameters and lengths can accommodate target vessels of various sizes. Second, the unique structural design makes the delivery flexible. Third, the low inflation pressure of the balloon for releasing the stent decreases the potential of target vascular damage. Finally, the trackability of this stent has improved compared with its congeners.

The stiffness of the traditional covered stents causes their difficult delivery to the tortuous intracranial vasculature (19, 22), and delivery failure has also been encountered (30). The Willis system is more flexible, which makes delivery easier. In Li's series, all stents were successfully delivered to the target vessel without apparent resistance (21). In our series, of the 19 stents, apparent delivery resistance was only encountered in two (10.5%, 2/19) patients, and no delivery failure was encountered, indicating the easy delivery for this system. Furthermore, the abundance of stent version provides the feasibility of selecting the minimal size of the stent for the target lesion, which contributes to the easy delivery. The relatively younger age (30.9 ± 12.6 years) in our series also contributes to the easy delivery.

Endoleak is another major concern for covered stents. In a small series that enrolled six patients with internal carotid artery injuries, endoleak occurred in all procedures (30). In the initial report for Willis stent, immediate endoleak occurred in 50% (4/8) of the patients (21). In our series, endoleak occurred in seven (36.8%, 7/19) patients immediately after the first balloon inflation, which was slightly lower than the previous report (21). Many possible reasons for endoleak have been proposed, including parent vessel spasms, size mismatch between the stent and parent vessel, mismatch of the sectional shape, rupture of the covered membrane, and possible shortening of the membrane (20). Strategies in preventing and treating endoleaks include confirmation of their correct position by multiple control angiograms before stent deployment, selection of an oversized stent, re-inflation, or performing additional stent angioplasty (20). In our series, re-inflation sealed 85.7% (6/7) of endoleaks and decreased the overall endoleak rate from 36.8 to 5.3%. A similar result was reported by other authors, in which 50% (2/4) of the endoleaks disappeared after a second inflation, resulting in an overall postoperative endoleak rate of 25% (2/8) (21). Minor endoleaks may disappear spontaneously during follow-up (20, 30). In our series, one patient suffered from endoleak after the operation, and the endoleak disappeared spontaneously during follow-up (Patient No. 10, illustrative Case 4, Figure 4). No further overlapped stent was needed in our series. In the seven cases with endoleak after the initial stent deployment, six (31.8%, 6/19) were minor, and only one (5.3%, 1/19) was apparent (Patient No. 2). We emphasize that minor endoleak in pseudoaneurysm may not indicate less dangerous than apparent endoleak. The delayed filing of the aneurysm may partially result in the absence of outlets, which is different from CCFs. Although the endoleak is minor, the patient may still at high risk of rebleeding because of the fragile aneurysm wall and subsequent dual anti-platelet regime. In our opinion, re-inflation is strongly recommended to seal the endoleaks. If an endoleak persists during the follow-up period, then placement of new covered stents or other bare stents may be an option (20, 21).

The relatively high thrombogenicity than bare stent is another shortcoming for covered stents (25). The relatively small series and lack of long-term angiographic follow-up indicate that the efficacy and durability of these devices were unreliable (19, 21). In our series, an intraoperative thrombogenic event occurred in one patient (Patient No. 13, 5.3%). In this patient, intravenous tirofiban hydrochloride successfully recanalized the occluded A1 segment. No intraoperative embolic events were reported in the previous study with Willis stent (20, 21). In a mean angiographic follow-up of 9 months, one patient encountered in-stent occlusion (Patient No. 4, 5.3%), and one patient encountered slight in-stent stenosis (Patient No. 8, illustrative case 3, Figure 3), which was indirectly indicated by the minimal distal flow on CT angiography. In the patient that encountered in-stent occlusion, insufficient dual antiplatelet therapy may be the primary reason. In the previous study with Willis covered stent, at 3 and 6 month angiographic follow-up, no evident in-stent stenosis was reported. At the 12 month angiographic follow-up, in-stent stenosis was not reported (20). Therefore, long-term follow-up is needed to elucidate the durability and efficacy of this device.

The coverage of vital perforators is also a major consideration for using a covered stent (2). Considering that only a few vital arterial branches originate from the trunk of ICA, such as the anterior choroidal artery and predominant posterior communicating artery (27), covered stents are reasonable for the internal carotid artery. The diversity of the Willis covered stent with different lengths further guaranteed complete coverage of the aneurysm orifice and preservation of vital perforators. Both in the previous and present reports, no perforator-related complications were encountered (20, 21).

Severe epistaxis, unilateral loss of vision, and cranial base fracture have been reported as the typical triad signs for traumatic pseudoaneurysms (7). Delayed and repeated epistaxis after head and facial trauma is the distinctive manifestation in most cases (6, 8, 20, 22, 27). However, in our series, only two patients (10.5%, 2/19) initially presented with epistaxis, and the majority (89.5%, 17/19) of patients presented vision loss as their chief complaint. This difference may be related to the unique patient population of our institution, which is a large center specialized in ophthalmology. During the clinical follow-up (median 11 months, ranging from 6 to 48 months), no recurrence of epistaxis was encountered, indicating the efficacy of this technique in repairing the vascular defect. Only 17.6% (3/17) of patients who initially presented with vision loss gained clinical improvement, and 82.4% (14/17) had no changes, indicating the poor recovery after direct optic nerve injury. Some authors have proposed that direct force to the frontal and lateral part of the orbit is prone to cause a traumatic pseudoaneurysm (26), as demonstrated by Patient No. 10 (illustrative case 4) in our series.

### Limitations

This study has some limitations. First, although this study was the largest pseudoaneurysm series treated with covered stents, the volume was relatively small, and all the cases were from a single medical institution. Second, the follow-up time was relatively short. Third, most (73.7%, 14/19) patients in our series only received CT angiography follow-up, and DSA was preferred to precisely evaluate the existence of a minor endoleak and the degree of in-stent stenosis. Four, the retrospective design failed to compare the outcome with other treatment strategies. Finally, no antiplatelet response was tested to guide the regime of antiplatelet therapy, and the great variance of antiplatelet responses among individuals would leave the patients at risk of thrombotic or hemorrhagic events.

## Conclusions

Despite the limitations, our initial results still demonstrated the feasibility, efficacy, and durability of using Willis covered stent in treating intracranial pseudoaneurysm of the internal carotid artery.

## Data Availability Statement

The original contributions presented in the study are included in the article/supplementary material, further inquiries can be directed to the corresponding author/s.

## Ethics Statement

The studies involving human participants were reviewed and approved by Ethic committee of Tongren Hospital. The patients/participants provided their written informed consent to participate in this study. Written informed consent was obtained from the individual(s), and minor(s)' legal guardian/next of kin, for the publication of any potentially identifiable images or data included in this article.

## Author Contributions

All authors listed have made a substantial, direct and intellectual contribution to the work, and approved it for publication.

## Conflict of Interest

The authors declare that the research was conducted in the absence of any commercial or financial relationships that could be construed as a potential conflict of interest.

## Abbreviations

CT: computed tomography
DSA: digital subtraction angiography
CCF: carotid-cavernous fistula.
